# Thresholds for statistical and clinical significance in systematic reviews with meta-analytic methods

**DOI:** 10.1186/1471-2288-14-120

**Published:** 2014-11-21

**Authors:** Janus Christian Jakobsen, Jørn Wetterslev, Per Winkel, Theis Lange, Christian Gluud

**Affiliations:** Rigshospitalet, Copenhagen Trial Unit, Centre for Clinical Intervention Research, Department 7812, Copenhagen University Hospital, Copenhagen, Denmark; Emergency Department, Holbæk Hospital, Holbæk, Denmark; Department of Biostatistics, Faculty of Health Sciences, University of Copenhagen, Copenhagen, Denmark

## Abstract

**Background:**

Thresholds for statistical significance when assessing meta-analysis results are being insufficiently demonstrated by traditional 95% confidence intervals and *P*-values. Assessment of intervention effects in systematic reviews with meta-analysis deserves greater rigour.

**Methods:**

Methodologies for assessing statistical and clinical significance of intervention effects in systematic reviews were considered. Balancing simplicity and comprehensiveness, an operational procedure was developed, based mainly on The Cochrane Collaboration methodology and the Grading of Recommendations Assessment, Development, and Evaluation (GRADE) guidelines.

**Results:**

We propose an eight-step procedure for better validation of meta-analytic results in systematic reviews (1) Obtain the 95% confidence intervals and the *P*-values from both fixed-effect and random-effects meta-analyses and report the most conservative results as the main results. (2) Explore the reasons behind substantial statistical heterogeneity using subgroup and sensitivity analyses (see step 6). (3) To take account of problems with multiplicity adjust the thresholds for significance according to the number of primary outcomes. (4) Calculate required information sizes (≈ the *a priori* required number of participants for a meta-analysis to be conclusive) for all outcomes and analyse each outcome with trial sequential analysis. Report whether the trial sequential monitoring boundaries for benefit, harm, or futility are crossed. (5) Calculate Bayes factors for all primary outcomes. (6) Use subgroup analyses and sensitivity analyses to assess the potential impact of bias on the review results. (7) Assess the risk of publication bias. (8) Assess the clinical significance of the statistically significant review results.

**Conclusions:**

If followed, the proposed eight-step procedure will increase the validity of assessments of intervention effects in systematic reviews of randomised clinical trials.

**Electronic supplementary material:**

The online version of this article (doi:10.1186/1471-2288-14-120) contains supplementary material, which is available to authorized users.

## Introduction

Systematic reviews summarise the results from randomised clinical trials. Meta-analysis is the main statistical method used in systematic reviews to analyse pooled results of trials [1, 2]. Some claim that results of systematic reviews should be considered hypothesis-generating and should primarily serve the purpose of designing future randomised clinical trials [3–5]. Others consider systematic reviews with meta-analysis the highest level of evidence assessing the effects of healthcare interventions [1, 2]. Studies have clearly shown that results of meta-analyses of trials with low risk of bias are more reliable than results of single large trials [6–11]. Inthout and colleagues quantified the error rates for evaluations based on single conventionally powered trials (80% or 90% power) compared to evaluations based on random-effects meta-analyses of a series of smaller trials [6]. When a treatment was assumed to have no effect but heterogeneity was present, the error rates for a single trial were increased more than 10-fold above the nominal rate [6]. Conversely, for meta-analyses on a series of trials, the error rates were correct [6]. When selective publication was present, the error rates were always increased, but they still tended to be lower for a series of trials than in a single trial [6]. It also appears intuitively evident that inclusion of all used data from all randomised clinical trials ever conducted shall be treated as a higher level of evidence compared to the data from only a single trial [2, 11–15]. We acknowledge that a systematic review with meta-analysis cannot be conducted with the same scientific cogency as a randomised clinical trial with pre-defined high-quality methodology addressing an *a priori* and quantitatively hypothesised intervention effect. Systematic review authors often know some of the eligible randomised clinical trials before they prepare their protocol for the systematic review, and hence, the review methodology is partly data driven. Nevertheless, understanding the inherent methodological limitations of a systematic review should lead to minimisation of these methodological limitations and optimisation of the remaining review methodology, which is the objective of this paper.

We recently described an operational five-step procedure for valid assessment of statistical and clinical significance in a single randomised clinical trial [12]. We will now, in a comparable manner, describe an eight-step procedure for a more valid assessment of results of systematic reviews of randomised clinical trials. Our procedure is based on and designed to be an extension to The Cochrane Collaboration Handbook and the Grading of Recommendations Assessment, Development, and Evaluation (GRADE) principles [13, 16–19]. The eight-step procedure can be used as part of a planned systematic review methodology or can be used to assess the validity of results from already published systematic reviews [20].

The following eight sections of the manuscript will correspond to each step of the proposed procedure.

## Methods

### Step 1: meta-analysis, the 95% confidence interval, and the *P*-value

In a meta-analysis, a summary statistic is calculated for each included trial, describing the observed intervention effect [13]. Then, an aggregated intervention effect estimate is calculated as a weighted average of the intervention effects estimated from the individual trials [13]. Review authors should always report both the confidence interval and the corresponding exact *P*-value from all meta-analyses. The confidence interval will show the range of uncertainty (considering the chosen threshold for statistical significance) around the aggregated intervention effect estimate. The *P*-value will show the probability of obtaining the observed or even a larger difference in intervention effect (disregarding possible bias) assuming that the null hypothesis is true (the null hypothesis implies that there is no difference in effect between the compared interventions) [12].

In a fixed-effect meta-analysis, the underlying assumption is that all of the included trials estimate the same intervention effect, i.e., differences in observed effects across trials are assumed to be caused by random error (‘play of chance’) [13]. In a random-effects meta-analysis, the underlying assumption is that the included trials do not estimate the same intervention effects – it is assumed that the estimates of individual trial intervention effects follow a normal or a log normal distribution [13]. The most commonly used random-effects model is the DerSimonian and Laird model [21]. However, the Hartung-Knapp-Sidik-Jonkman random-effects model assuming a t-distribution of log (RR) (for dichotomous outcomes) seems to be a more valid meta-analysis method [22]. It is often likely that a given intervention will have different effects across the included trials depending on different forms of the interventions, different definitions of the outcomes, different types of included participants, etc. The random-effects model assumption will, therefore, often be more realistic than the fixed-effect model assumption [13]. If there is absence of statistical heterogeneity (the between trial variance of the estimated intervention effects is close to zero [23]), then the fixed-effect and the random-effects models will show identical results [13]. If there is substantial statistical heterogeneity, the fixed-effect meta-analysis will, in some circumstances, show erroneous results because the between trial variance is not appropriately accounted for. In such a case, the random-effects meta-analysis result should be regarded as the main result. On the other hand, if one or two trials accounts for approximately 80% or more of the total weight in a fixed-effect meta-analysis, then the random-effects meta-analysis might show erroneous results because the larger trials with the greatest precision are inappropriately down-weighted [24]. In such a case, the fixed-effect meta-analysis result should be regarded as the main result. We recommend always reporting results from both fixed-effect and random-effects meta-analyses. If the fixed-effect and the random-effects meta-analyses show different results, then the most conservative result (the analysis with the highest *P*-value) should be chosen as the main result [13]. Choosing the most conservative result will take account of the mentioned pitfalls of the two analyses [13]. Substantial discrepancies between the results of the two methods should be reported and discussed thoroughly (see **step 2**).

### Step 2: investigating statistical and clinical heterogeneity

Implications of clinical and statistical heterogeneity should always be considered when meta-analyses are conducted [13, 25, 26]. Substantial statistical heterogeneity may be identified by visual inspection of forest plots and by the calculated statistical heterogeneity (for example, I^2^ or D^2^) [13, 25, 26]. It must be noted that the statistical heterogeneity (both I^2^ and D^2^) will increase by including trials with large sample sizes because the variance of the trials’ intervention effects will decrease with the increased sample size and event size. As a result, the ratio of the between trial variance to the total variance will also increase. In other words, small and clinically irrelevant intervention effect differences across trials might lead to significant statistical heterogeneity when confidence intervals of the trial intervention effects are narrow [27]. Statistical heterogeneity should always be interpreted with caution but especially when analysing large sample sizes [21]. Underlying reasons behind significant statistical heterogeneity in meta-analyses should be investigated by assessing trial characteristics in subgroup analyses and sensitivity analyses (see step 6), and it should be reconsidered if all of the included trials should be included in the analyses. For example, if the statistical heterogeneity seems to be caused by different forms of the intervention or a different assessment procedure, then the need for excluding some of the trials from the main analysis should be considered. If any trial is excluded from any analysis, then the reasons for excluding the trial should be clearly reported in the manuscript.

### *Post hoc*analyses

Ideally all analyses in a systematic review should be planned at the protocol stage, but *post hoc* analyses might be warranted if unexpected clinical or statistical heterogeneity is identified during the analysis of the review results. Nevertheless, *post hoc* analyses should always be interpreted with great caution and it should be made very clear, which analyses were pre-defined in the published protocol and which were not. *Post hoc* analysis should be regarded as exploratory and hypotheses generating.

### Step 3: problems with multiplicity due to multiple outcomes

The overall risk of falsely rejecting the null hypothesis for at least one outcome (the family-wise error rate) will increase with the number of outcome comparisons [28, 29]. Problems with multiplicity in systematic reviews have major implications for the interpretation of the confidence intervals and the *P*-values [29, 30] – and problems with multiplicity are often not accounted for in systematic reviews [28, 29, 31]. For example, thresholds for significance in meta-analyses are rarely adjusted if more than one primary outcome is used [13].

Most systematic reviews will include multiple outcome comparisons, and if review authors are free to choose and highlight single results among the many comparisons, there will be an increased risk of false declaration on the effectiveness of an assessed intervention. Data driven *post hoc* analyses will be avoided if the review methodology is clearly pre-defined and not changed during the analysis of the review results. A straightforward way to deal with some of the multiplicity problems is to publish a protocol before the literature search begins (for example, at PROSPERO (http://www.crd.york.ac.uk/PROSPERO/)) [13, 31–33]. In the protocol, the statistical methodology should be described in detail, including a clear definition of the primary, secondary, and exploratory outcomes [13, 31–33]. Proper adjustments according to problems with multiplicity (see below) should be estimated based on the outcome hierarchy specified in the protocol. The main conclusions of the review ought to be based on the results on the primary outcome/s. Hence, adjustments due to problems with multiplicity may also be limited to the primary outcome/s. This will in itself limit the risk of type I error and will make the threshold adjustments simpler and practically feasible. If an outcome is assessed a multiple number of times, then either the thresholds for significance should be adjusted accordingly or the time point of primary interest should be pre-specified.

A systematic review should summarise all available evidence for a given medical intervention and choosing only one primary outcome will often be too restrictive. In advance, it will often be unknown, which outcomes the eligible trials have used and it is important to assess both beneficial and harmful intervention effects [5]. It is, therefore, often advisable to use more than one patient-relevant primary outcome in a systematic review. The Cochrane Collaboration recommends using up to three primary outcomes – for example, all-cause mortality, serious adverse events, and quality of life [13]. The use of more than one primary outcome (co-primary outcomes) necessitates adjustments of the thresholds for significance because of problems with multiplicity [34].

Different statistical methods have been proposed to adjust confidence intervals and *P*-values when multiple outcome comparisons are used [12]. Most adjustment methods have focused on adjustments of the *P*-value threshold, but adjusted confidence intervals can often be calculated based on an adjusted *P*-value and an effect estimate [12, 35, 36]. There is an extensive statistical literature about problems with multiplicity in observational studies and randomised clinical trials [29], but problems with multiplicity in systematic reviews have received limited attention [29, 31]. Some methods have been developed to deal with problems with multiplicity in systematic reviews [37–39], but no simple and completely satisfactory solution to the problem of multiple comparisons in systematic reviews has been developed yet [29, 31].

The Bonferroni procedure divides the specified *P*-value threshold (for example, 0.05) with the number of outcome comparisons and this method can be used to control the family-wise error rate [34]. If it is plausible that there is no correlation between multiple primary outcomes, then Bonferroni adjustment may be used. However, the Bonferroni procedure is mostly a too conservative adjustment method as most outcomes are interdependent (for example, all cause mortality and serious adverse events will often be positively correlated outcomes) [34]. To calculate more precise adjustments of the thresholds for significance in systematic reviews, an estimation of the correlation between the co-primary outcomes will be needed. Such a correlation will often be unknown and erroneous assumptions about correlations might lead to erroneous results. Because the ‘true’ multiplicity adjusted thresholds for significance lie somewhere between the unadjusted threshold (for example, 0.05) and the Bonferroni adjusted threshold, we suggest a pragmatic approach. We suggest dividing the pre-specified *P*-value threshold with the value halfway between 1 (no adjustment) and the number of primary outcome comparisons (Bonferroni adjustment). This will result in a multiplicity adjusted threshold using 1 primary outcome = 0.05, 2 primary outcomes = 0.033, and 3 primary outcomes = 0.025.

The use of full Bayesian statistics can account for problems of multiplicity due to multiple testing [24, 40, 41]. However, this use would imply integration of complicated models and software for analysing the review results [24, 40, 41] and would entail the need for specifying multiple prior distributions, which has its own problems [12, 24, 40, 41].

### Step 4: trial sequential analysis

#### Required information size

If the sample size has not been reached in a randomised clinical trial, then the threshold for statistical significance ought to be adjusted [12, 42]. A similar methodology should apply to a meta-analysis that does not reach a required information size (≈ the *a priori* required number of participants for a meta-analysis to be conclusive) [25, 26]. The 95% confidence interval may show the range of uncertainty of the observed intervention effect estimate and may improve the interpretability of meta-analysis result. However, reporting results from meta-analyses without linking the confidence intervals, the intervention effect estimates, and the *P*-values to an estimated required information size is erroneous for a number of reasons:

- Most review authors do not assess if an accrued information size is sufficient or not to detect or reject a given intervention effect, and this is problematic as nearly all meta-analyses in systematic reviews are underpowered (the meta-analyses do not have enough information to reject or accept the null hypothesis when the null hypothesis is false) [25, 26, 43]. Without an estimation of a required information size it is difficult to interpret an apparent neutral (no significant difference) meta-analysis result – it becomes unclear whether a neutral meta-analysis result indicates that there is no difference in effect between the compared interventions, or if the result indicates that the information size is too small to demonstrate or discard the anticipated intervention effect [11]. Furthermore, meta-analyses with too small accrued information size (sparse data) have an increased risk of either overestimating or underestimating the effect size and variance [25, 26, 44–46]. Therefore, without a required information size it will also be difficult to interpret a meta-analysis result indicating a difference in effect, i.e., an observed difference in effect might be caused by the random error due to the low information size.
- There should be a low risk of turning a significant (for example, a *P*-value below 0.05) meta-analysis result into an insignificant result when future trials are included – ‘unstable’ meta-analysis results should be avoided for obvious reasons. It has been shown that a statistically significant meta-analysis based on too low information sizes, often at a later time point, will change from statistically significant to statistically non-significant [25, 26, 47].
- The Cochrane Collaboration recommends that all systematic reviews are updated at least every second year [13], and there might be a lower chance of a review being updated if a meta-analysis shows significant results. If systematic review authors are allowed to assess statistical significance each time the review is updated without adjusting the level of statistical significance, premature declaration of effects will ensue and the risk of falsely rejecting the null hypothesis will increase (see also **step 3** for a description of other problems with multiplicity) [14, 25, 26].

It is, therefore, of importance to estimate a required information size before conducting a meta-analysis. To estimate a required information size, it is necessary:

- To estimate an anticipated intervention effect, i.e., to define a hypothesis alternative to the null hypothesis (for example, a mean difference, an odds ratio, or a hazard ratio [13, 48]). This hypothesised difference in effect between the compared intervention groups should be based on the most realistic intervention effect as suggested by prior evidence (for example, results from former randomised clinical trials, or known effects from other similar interventions [49–51]). As supplementary analyses, the point estimate from the meta-analysis on the outcome and the limit of the 95% confidence interval closest to no effect can be used as anticipated intervention effects.
- To estimate a variance of the anticipated difference in intervention effect (for example, a standard deviation for a continuous outcome or a proportion of control participants with an event for a dichotomous outcome). Ideally this variance should be based on results from empirical data, for example, former systematic reviews, former randomised clinical trials, or large observational studies.
- To estimate a variance of the intervention effect estimates between trials (D^2^ = the percentage that the between-trial variability constitutes of the sum of the between-trial variability and a sampling error estimate considering the required information size) [25, 26, 52]. If a random-effects meta-analysis is chosen as the one of primary interest, it may be pre-defined that the observed between trial variance (empirical variance) will be used to calculate the required information size [47, 52]. However, if the observed heterogeneity is zero, it may not be unwise to use a heterogeneity of 25% [47]. If a fixed-effect meta-analysis is chosen as the one of primary interest, then the variance between trials should be zero (see **step 1**).
- To decide on an acceptable risk of falsely rejecting the null hypothesis (alpha or type I error). As we have described in **step 3,** to adjust the risk of type I error according to the number of outcome comparisons we suggest dividing a pre-specified *P*-value threshold with the value halfway between 1 and the number of primary outcome comparisons.
- To decide on an acceptable risk of falsely confirming the null hypothesis (beta or type II error). We recommend 10% or 20%.

Required information sizes can easily be calculated using the program trial sequential analysis which together with a user manual can be downloaded for free at our website (http://www.ctu.dk/tsa/) [47]. The diversity-adjusted required information size should be reported for all primary and secondary outcomes in the protocol.

Review authors might be tempted to calculate the diversity-adjusted required information size based on unrealistically large anticipated intervention effects – large anticipated intervention effects lead to small required information sizes and the thresholds for significance will be less strict after the information size has been reached [25, 26]. This problematic incentive for using too large anticipated intervention effects to reduce the required information size might be counterbalanced by the use of a simple Bayes factor (see **step 5**).

### Methods to adjust thresholds for significance if the required information size has not been reached

Trial sequential analysis [25, 26] or another valid sequential method [53] may be used to show if information size adjusted thresholds for significance are crossed or not. Trial sequential analysis uses the Lan-DeMets trial sequential monitoring boundaries based on a O’Brien-Fleming alfa-spending function because the sample sizes of the included trials vary. The monitoring boundaries for benefit, harm, or futility show the adjusted thresholds for significance (adjusted thresholds of the confidence intervals and the *P*-values) if a required information size has not been reached [25, 26, 47, 54]. We recommend analysing all primary and secondary outcome comparisons with trial sequential analysis [25, 26, 47, 54] or another valid sequential method [25, 26, 53].

### Fixed-effect and random-effects trial sequential analysis

The trial sequential analyses should be performed using both a fixed-effect and a random-effects model [14, 25, 26]. Analogous to the meta-analysis (see **step 1**), the trial sequential analysis with the highest *P*-value should be chosen as the primary. If a fixed-effect trial sequential analysis is of primary interest, then the variance between trials should be zero because the underlying assumption behind the fixed-effect model is that all included trials are estimating the same intervention effect. This also means that the calculation of the required information size should not be adjusted according to variance between trials if a fixed-effect meta-analysis is chosen as the one of primary interest.

### If the diversity-adjusted required information size is reached or surpassed

If the diversity-adjusted required information size is reached, then the traditional thresholds for significance (95% confidence intervals not containing 1.00 for binary outcomes or 0.00 for continuous outcomes, and a corresponding *P*-value under 0.05) may be used unless these thresholds are adjusted according to problems with multiplicity (see **step 3**).

### Step 5: Bayes factor

A low *P*-value indicates that an observed result is unlikely given the null hypothesis is true [12] – the P-value relates to the probability that there is no difference in effect between the compared interventions. Even a low *P*-value from a meta-analysis can be misleading if there is also a low probability that data are compatible with the anticipated intervention effect (see **step 4**). In other words, the probability that the actual measured difference in effect of the compared interventions resulted from an a priori anticipated ‘true’ difference needs to be considered. For this purpose it is helpful to calculate Bayes factors for the primary outcome/s (http://www.ctu.dk/tools-and-links/bayes-factor-calculation.aspx) [55, 56]. Bayes factor is the ratio between the probability of the meta-analysis result given the null hypothesis (H_0_) is true divided by the probability of the meta-analysis result given the alternative hypothesis (H_A_) is true [12]. In the following, we have chosen to define the alternative hypothesis (H_A_) as the anticipated intervention effect used in the calculation of the required information size in the trial sequential analysis (see **step 4**) [12], but Bayes factor can be defined differently [12]. Bayes factor as defined here may be calculated via the following formula (http://www.ctu.dk/tools-and-links/bayes-factor-calculation.aspx):


μ_A_ = the intervention effect hypothesised in the estimation of the required information size (for example, a mean difference, a log odds ratio, a log relative risk, or a log hazard ratio).

 = the intervention effect shown by the meta-analysis result (for example, a mean difference, a log odds ratio, a log relative risk, or a log hazard ratio). SE  = standard error of . Details about Bayes factor and relevant basic statistical methodology may be found elsewhere [12, 50].

**Figure 1:**
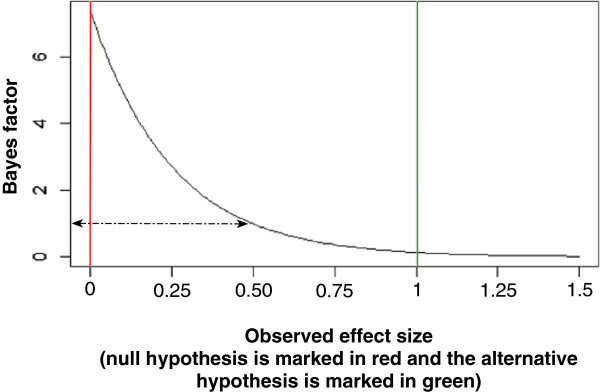
**A figure showing how Bayes factor will change according to different observed effects.** The red left vertical line represents the null hypothesis (an effect of null), and the right green vertical line represents an alternative hypothesis to the null hypothesis with an effect of 1.0. The black curve shows that Bayes factor will be 1.0 when the observed effect size is exactly half of the effect size of the alternative hypothesis; and the curve shows that Bayes factor will decrease with increasing observed effect sizes.

A narrow confidence interval associated with a low *P*-value does not, as mentioned, necessarily correspond to a low Bayes factor – and a *P*-value less than 0.05 may therefore, in some circumstances, misleadingly indicate evidence for an intervention effect [50, 55]. A *P*-value less than 0.05 combined with a Bayes factor greater than 1.0 obtained from the observed data render more credit to the null hypothesis being true than the alternative hypothesis being true. A high Bayes factor will indicate that a meta-analysis result should be interpreted with caution, or, at least, indicate that the meta-analysis result is produced by an intervention effect that is lower than the anticipated intervention effect. A low Bayes factor together with a low *P*-value will correspond to a high probability of an intervention effect similar to or greater than the anticipated intervention effect used in the calculation of the required information size (Figure 1).

### The Bayes factor threshold for significance

A Bayes factor less than 0.1 (a tenfold higher likelihood of compatibility with the alternative hypothesis than with the null hypothesis) may be chosen as threshold for significance [50]. However, spurious intervention effects caused by sparse data (see **step 4**) will also affect Bayes factor. Hence, if the required information size has not been reached, then even a Bayes factor less than 0.1 should be interpreted with caution [12, 57].

As mentioned in **step 4**, to reduce the required information size there is a risk of review authors using unrealistically large anticipated intervention effects in the calculation of the required information size. This problematic incentive will be counterbalanced by the use of Bayes factor because Bayes factor will increase if the meta-analysis shows an intervention effect smaller than the anticipated intervention effect (Figure 1). In other words, if unrealistically large intervention effects are anticipated, then observed unbiased data will often show smaller and more realistic intervention effects which will result in a relative high Bayes factor. The use of Bayes factor might be an incentive for a more realistic and smaller estimation of anticipated intervention effects, which will generally increase the validity of meta-analysis results and decrease the risk of meta-analysis, either overestimating or underestimating intervention effects. However, Bayes factor will still be misleading when an unrealistically large anticipated intervention effect is confirmed by ‘play of chance’, by an unrealistically large observed intervention effect [12, 50].

### Step 6: the potential impact of systematic errors (‘bias’) on the meta-analysis results

#### Overall risk of bias

We have in step 3 to 5 described how to assess and take account of the risk of random error. However, it is of utmost importance also to assess the risk of systematic error (‘bias’) through subgroup analyses and sensitivity analyses [13]. Empirical evidence has repeatedly shown that trials with high risk of bias tend to overestimate benefits and underestimate harms [58–64]. The bias risk domains generation of allocation sequence, allocation concealment, blinding of participants and treatment providers, blinding of outcome assessors, incomplete outcome data (see paragraph below), selective outcome reporting, and industry funding have been shown to be of particular importance [13, 58–64]. A randomised clinical trial should, therefore, only be classified as overall ‘low risk of bias’ if all of the above mentioned bias components are assessed as ‘low risk of bias’ [13, 58–64]. The main conclusion of the systematic review ought to based on results of trials with low risk of bias – so, such an analysis should always be presented. A subgroup analysis should always be performed comparing the effects of trials with ‘low risk of bias’ to trials with ‘high risk of bias’.

### The range of uncertainty due to the missing outcome data

If trial investigators have not used valid methods (for example, multiple imputation) to deal with missing data in the included randomised clinical trials [13], then there is a risk of biased review results [20, 65]. For example, if a certain group of participants are systematically lost to follow-up in only one of the compared intervention groups, then the review results might show a difference in effect between the compared groups due to attrition bias. If individual patient data are available, then multiple imputation might be used by the review authors, but even multiple imputation might lead to biased results if data are ‘missing not at random’ [65, 66].

For all meta-analyses, we recommend using at least two sensitivity analyses to assess the potential impact of the missing outcome data (risk of attrition bias) on the meta-analysis results [67]. The first sensitivity analysis is a ’best-worst-case’ scenario where it is assumed that all participants lost to follow-up in the experimental group have had a beneficial outcome (for example, had no serious adverse event); and all those with missing outcomes in the control group have had a harmful outcome (for example, have had a serious adverse event). The second sensitivity analysis is a ’worst-best-case’ scenario where it is assumed that all participants lost to follow-up in the experimental group have had a harmful outcome; and that all those lost to follow-up in the control group have had a beneficial outcome. If continuous outcomes are used, then a ‘beneficial outcome’ might be the group mean plus 2 standard deviations (or 1 standard deviation) of the group mean, and a ‘harmful outcome’ might be the group mean minus 2 standard deviations (or 1 standard deviation) of the group mean (see Additional file 1).

The results from both of these two extreme scenarios will most likely be unrealistic. However, these sensitivity analyses show the range of uncertainty due to missing data. The primary meta-analysis result may then be related to the results from the sensitivity analyses. If the primary meta-analysis result and the sensitivity analyses show similar confidence intervals and *P-*values, then the validity of the review results will increase considerably. If the primary meta-analysis results differ substantially from the results of the sensitivity analyses, then this will show that there is a risk of biased results due to attrition bias.

### Step 7: publication bias

Systematic reviews aim to identify and include all randomised clinical trials addressing the question of the review [13]. However, trials with certain results might not be published for different reasons and this might consequently bias the review results [13]. Funnel plots can assess the risk of such ‘publication bias’ together with other bias [13].

A funnel plot is a simple scatter plot with the intervention effect estimates from the included trials (odds ratios and risk ratios should be plotted on a logarithmic scale) on the horizontal scale and the standard error of the intervention effect estimate on the vertical scale [13]. For example, if smaller trials without statistically significant effects remain unpublished, this will lead to an asymmetrical appearance of the funnel plot with a gap in the corner of the plot [13]. It is evident that such publication bias may tend to overestimate intervention effects [68].

Funnel plot should only be used to assess the risk of bias if at least 10 trials are included in the meta-analysis [13]. Funnel plots cannot be used to assess the risk of publication bias if the included trials are of similar size and some effect estimates are naturally correlated with their standard errors and therefore can produce spurious asymmetry in funnel plots [69]. Other types of bias or ‘true’ heterogeneity of the trial intervention effects might also produce asymmetry [13]. Contour lines corresponding to ‘milestones’ of statistical significance (for example, *P* = 0.05) can be added to the funnel plot and may help to differentiate between funnel plot asymmetry caused by publication bias and other factors [70]. For example, if trials appear to be missing in areas of statistical non-significance, then this may indicate that the asymmetry is due to publication bias. On the other hand, if the supposed missing trials are in areas of higher statistical significance, then the cause of the asymmetry may be due to factors other than publication bias. If there are no trials showing statistically significant effects, then publication bias may not be a plausible explanation for funnel plot asymmetry [13, 71].

A number of tests can assess funnel plot asymmetry (for example, Egger, Deeks, Harbourd, Begg) [13]. These tests should be interpreted in the light of visual inspection of the funnel plot and the results from these tests should be interpreted with caution. In general, the proposed tests have relatively low power [13].

The ‘trim and fill’ method can be used to assess the robustness of the review results. The trim and fill method aims to identify and correct funnel plot asymmetry arising from publication bias [13, 72]. The method removes the smaller trials causing funnel plot asymmetry, use the ‘trimmed’ funnel plot to estimate the true ‘centre’ of the funnel, and then includes both the removed trials and their ‘missing’ counterparts around the centre. The trim and fill method then provides an estimate of an intervention effect estimate adjusted for the assumed publication bias and the ‘missing’ trials are plotted. The trim and fill method assumes that the funnel plot asymmetry is caused by publication bias, but other factors (for example, heterogeneity) will often also cause or contribute to an asymmetry [13]. Therefore, the results from the trim and fill methods should be interpreted with caution and should primarily be hypothesis generating.

We have in this paragraph summarised The Cochrane Collaboration methodology to assess the risk of publication bias. For a more detailed description of assessments of publication bias, please consult The Cochrane Handbook for Systematic Reviews of Interventions [13].

### Step 8: statistical significance and clinical significance

It will be impossible to interpret any review result without a thorough assessment of statistical significance and step 1 to 7 has described how to assess statistical significance. However, more than statistical significance is required before an intervention is declared as being effective, i.e., the size of the intervention effect must be clinically relevant in addition to the statistical significance. For clinically relevant outcomes, such as mortality, it is difficult to define a lower threshold for clinical significance. Any prevention, whatever small, of patient-important outcomes may seem relevant [11, 12]. The clinical implications of statistically significant results on surrogate outcomes (‘indirectness’, in the GRADE system [16]), for example, serum levels of cholesterol or virological response, can often be questioned even with *P*-values far below 0.05 [11, 12]. Regardless of the type of outcome, small beneficial intervention effects will often not have any clinical relevance if adverse effects are taken into consideration [11, 12]. Even rare serious adverse effects may rule out the rational use of an otherwise beneficial intervention [73]. To assess the clinical significance of intervention effects it is important to perform a thorough assessment of the balance between beneficial and harmful effects [11, 12]. It is also important to relate the trial participants to a clinical population. Clinical implications of review results cannot be extrapolated to patient groups other than the patients included in the review [11]. Moreover, if a new intervention shows statistically significant effects but the size of the intervention effect is smaller compared to another intervention, then the new intervention effect might be considered as not clinically significant.

To avoid erroneous interpretations, assessment of the clinical significance of an intervention effect should only be assessed if statistical significance has been obtained, i.e., that the prior seven steps of our procedure have shown indications of a statistically significant result [74]. On the other hand, if statistical significance has been reached, then clinical significance must be assessed [11, 12, 16, 73, 74].

Preparing summary of findings tables according to the GRADE guidelines is an excellent way to demonstrate the clinical implications of review results [16–19] (Table 1). Reporting confidence intervals, ‘minimal clinical relevant differences’ for continuous outcomes, numbers-needed-to-treat for binary outcomes, and median survival times for survival data may also improve the clinical interpretability of review results [2, 11, 12, 16–19].

**Table 1:** **Quality assessment criteria according to GRADE adopted after reference** [16]

Study design	Levels of confidence in estimate	Decrease confidence estimate if	Increase confidence estimate if
Randomised clinical trials	High	**Risk of bias**	**Large intervention effect**
		One level if serious	One level if large
		Two levels if very serious	Two levels if very large
	Moderate	**Imprecision**	**Dose response**
		One level if serious	One level if evidence of dose response
		Two levels if very serious	
Observational studies	Low	**Indirectness**	**All plausible confounding**
		One level if serious	One level if confounding would reduce a demonstrated effect
		Two levels if very serious	
	Very low	**Heterogeneity**	**All plausible confounding**
		One level if serious	One level if confounding would suggest a spurious effect when results show no effect
		Two levels if very serious	
		**Publication bias**	
		One level if serious	
		Two levels if very serious	

### Recommendations

To assess the statistical and clinical significance of results from systematic reviews, we propose the following eight-step procedure:

1. Calculate and report the confidence intervals and *P*-values from all fixed-effect and random-effects meta-analyses. The most conservative result should be the main result.
2. Explore the reasons behind substantial statistical heterogeneity by performing subgroup analyses and sensitivity analyses (see step 6).
3. Adjust the thresholds for significance (*P*-values and the confidence intervals from the meta-analyses and the risks of type I error in the trial sequential analysis) according to the number of primary outcomes.
4. Calculate and report realistic diversity-adjusted required information sizes and analyse all primary and secondary outcomes in the review with trial sequential analysis. Report if the trial sequential monitoring boundaries for benefit, harm, or futility are crossed [25, 26]. The trial sequential analyses will adjust the confidence intervals and the thresholds for significance by relating the accrued data to the required information sizes [25, 26].
5. Calculate and report Bayes factor for the primary outcome/s based on a pre-specified anticipated intervention effect (same anticipated intervention effect as the one used to estimate the required information size) (http://www.ctu.dk/tools-and-links/bayes-factor-calculation.aspx). A Bayes factor less than 0.1 (a tenfold higher likelihood of compatibility with the alternative hypothesis than with the null hypothesis) may be used as threshold for significance.
6. Use subgroup analysis and sensitivity analyses to assess the potential impact of systematic errors (bias).
7. Assess the risk of publication bias (funnel plot).
8. Assess clinical significance of the review results if the prior seven steps have shown statistically significant results.

Table 2 summarises our suggestions for a more valid assessment of intervention effects in systematic reviews with meta-analytic methods, and we present an example of how the eight-step assessment can be used to assess statistical significance and clinical significance (see Additional file 1). For simplicity, we have only assessed the result of the primary outcome.

**Table 2:** **Suggestions for a more valid assessment of intervention effects in systematic reviews**

**Step 1**	Calculate and report the *P*-values and the 95% confidence intervals from all fixed-effect and random-effects meta-analyses. The most conservative result should be the main result.
**Step 2**	Explore the reasons behind substantial statistical heterogeneity by performing subgroup analyses and sensitivity analyses (see step 6).
**Step 3**	Adjust the thresholds for significance (*P*-values and the confidence intervals from the meta-analyses and the risks of type I error in the trial sequential analysis) according to the number of primary outcome comparisons.
**Step 4**	Calculate and report a realistic diversity-adjusted required information size and analyse all of the outcomes with trial sequential analysis. Report if the trial sequential monitoring boundaries for benefit, harm, or futility are crossed.
**Step 5**	Calculate and report Bayes factor for the primary outcome/s based on the anticipated intervention effect used to estimate the required information size (http://www.ctu.dk/tools-and-links/bayes-factor-calculation.aspx). A Bayes factor less than 0.1 (a ten-fold higher likelihood of compatibility with the alternative hypothesis than with the null hypothesis) may be chosen as threshold for significance.
**Step 6**	Use subgroup analysis and sensitivity analyses to assess the potential impact of systematic errors (‘bias’).
**Step 7**	Assess the risk of publication bias.
**Step 8**	Assess clinical significance of the review results if the prior seven steps have shown statistically significant results.

All of these aspects should be prospectively planned and published in the protocol for the systematic review before the literature search begins.

### The eight-step procedure, The Cochrane Collaboration methodology, and the GRADE system

The eight-step procedure is designed to specify and assess the *thresholds* for significance in systematic reviews – the overall systematic review methodology should always be based on The Cochrane Handbook for Systematic Reviews of Interventions [2, 13]. The GRADE system provides a valid assessment of the quality of evidence in systematic reviews [2, 16–19]. Most of the methodological elements of the eight-step procedure, for example, trial sequential analyses, have already been used in several systematic reviews (for example, [75, 76]) but are neither described in The Cochrane Handbook for Systematic Reviews of Interventions nor the GRADE system. The eight-step procedure summarises the necessary methodology to assess the thresholds for significance and adds to the Cochrane methodology and the GRADE system: adjustments of the thresholds for significance according to problems with multiplicity and small information sizes, estimations of required information sizes, best-worst and worst-best case scenarios to assess the potential impact of missing data, and a calculation of Bayes factor. Table 1 summarises how GRADE may be used to assess the quality of the evidence [16], and we present an overview in Table 3 of how trial sequential analysis (**step 4**) may be used as a supplement for a more thorough assessment of ‘imprecision’ [77].

**Table 3:** **How trial sequential analysis can supplement the assessment of GRADE for ‘imprecision’** [77]

Trial sequential analysis	Assessment of imprecision [77]
If none of the sequential boundaries for benefit, harm, or futility are crossed and the anticipated intervention effect is realistic.	The evidence should be downgraded two levels of quality according to imprecision (see Table 1).
If one of the boundaries for benefit, harm, or futility are crossed and the anticipated intervention effect is realistic.	The evidence should not be downgraded according to imprecision (see Table 1).
If the anticipated intervention effect is considered unrealistic.	The trial sequential analysis should be repeated using the limit of the confidence interval, closest to zero effect from the traditional meta-analysis as the anticipated intervention effect. If the sequential boundaries are crossed then the level of evidence should not be downgraded (see Table 1). If the sequential boundaries are not crossed, the trial sequential analysis should be repeated, this time, using the intervention effect estimate from the meta-analysis as the anticipated intervention effect. If the sequential boundaries are crossed, then the evidence should be downgraded one level of quality; if the sequential boundaries are not crossed, then the evidence should be downgraded two levels of quality (see Table 1).

## Discussion

We argue that a systematic review should be the prerogative and the incentive for conducting new trials and introducing new interventions into clinical practice. The systematic review ought be at the top of the hierarchy of evidence [6, 7, 11]. Nevertheless, there is a high risk of biased review results if reviews are not conducted with high methodological rigour. In order to avoid premature and erroneous conclusions, we have described the importance of valid thresholds for statistical and clinical significance in a systematic review, and we have also described a systematic step-wise methodological approach which will increase the methodological validity and quality of the interpretation of review results.

### Strengths and limitations

The proposed eight-step procedure has strengths and limitations.

The eight-step procedure has the strength that it: (1) summarises The Cochrane Collaboration methodology related to the specification and assessment of the thresholds for significance in systematic reviews; (2) systematically adjusts the thresholds for significance according to the number of primary outcome comparisons and the fraction of the required information size that has been reached; (3) provides a likelihood ratio of the probability that a meta-analysis result is compatible with the null hypothesis divided by the probability that the result is compatible with an anticipated intervention effect; (4) limits the incentives for review authors both to overestimate and underestimate the anticipated intervention effect (see **step 4** and **step 5**); (5) provides a more thorough assessment of the review results with a specific and elaborated evaluation of imprecision which may be used for a more accurate GRADE rating [16, 77]; and (6) forces investigators and consumers of systematically reviewed clinical research to judge clinical significance.

Our methodology has limitations.

First, our eight-step procedure is based on already well-established methodologies, but we lack large comparative studies comparing the use of the procedure to ‘usual practice’. We will address this issue in forthcoming articles.

Second, the pragmatic approach we recommend to be used for multiplicity adjustment (dividing 0.05 with the value halfway between 1 and the number of primary outcome comparisons) is not based on evidence. Our approach is based on the fact that the ‘true’ multiplicity adjusted threshold for significance lies between the unadjusted threshold and the Bonferroni adjusted threshold (see **step 3**). Nevertheless, most systematic reviewers do not adjust the thresholds for significance at all, which seems worse than both the conservative approach (Bonferroni adjustment) and our suggested pragmatic approach. As we have described in **step 3,** to calculate more precise adjustments of the thresholds for significance in systematic reviews an estimation of the correlation between the co-primary outcomes will be needed (see **step 3**). Such a correlation will often be unknown and erroneous assumptions about correlations might lead to erroneous results.

Third, the required information size, the trial sequential analysis, and the size of Bayes factor will all be highly dependent on the choice of the anticipated intervention effect which often will be difficult to quantify in advance. To reduce this limitation, it will often be warranted to perform sensitivity analyses using different estimations of the anticipated intervention effects. For example, as supplementary trial sequential analyses, the point estimate from the meta-analysis on the outcome and the limit of the 95% confidence interval closest to no effect can be used as the anticipated intervention effects; or an additional Bayes factor may be calculated using a smaller (‘sceptical’) anticipated intervention effect (for example, a relative risk halfway between the intervention effect anticipated in the calculation of the required information size and 1.0). The uncertainty related to the quantification of the anticipated intervention effects is a major limitation of our procedure as well as Bayesian analyses. To make the estimation of the anticipated intervention effects as optimal and as objective as possible, we recommend, as one option, to base the estimation of the anticipated intervention effect on former randomised clinical trials. If this is done, then the results from the trials used to estimate the anticipated intervention effects will probably be used again in the full review analysis, i.e., the estimation of the required information size take the form of an adaptive estimation [51, 78]. Hence, the risk of type I error will increase [51, 78]. Because of this risk of circular reasoning, ideally, it is, necessary to adjust the required information size by applying a penalty for the weight of data from former systematic reviews (or trials) [51, 78]. If the anticipated intervention effect estimate is based on, for example, an estimation of a ‘minimally clinically relevant’ intervention effect, then no adjustments will be needed. We acknowledge the theoretical need for such further adjustments, but then, the review analyses will become very complicated because the analysis methods would differ, depending on how the anticipated intervention effects are defined. Furthermore, our recommendations are already considerably tightening the thresholds for significance in systematic reviews, and if thresholds are too tight, there is a risk of ‘throwing the baby out with the bath water’.

The trial sequential analysis, the required information size, and Bayes factor may be influenced by *post-hoc* adjustments and erroneous quantifications of the alternative hypothesis, especially if they have not been declared transparently in a protocol published before the systematic review begins. If the anticipated intervention effects are clearly defined in a published review protocol, and if pre-defined sensitivity analyses assess the uncertainty of the estimation of the anticipated intervention effects, then many of the problems with using anticipated intervention effect will be limited. The uncertainty of the estimation of the anticipated intervention effect is a challenge and a major limitation – but remains a necessary evil. A required information size and adjusted thresholds for significance cannot be calculated without estimations of an anticipated intervention effect.

We are sure that our eight-step procedure will be met with critical comments. Our recommendations will lead to fewer interventions which seem to offer benefits, the introduction of effective interventions in clinical practice may be delayed, and patient populations may stay without evidence-based interventions. We agree with such risks, but all things considered we do argue that a conservative scenario is more ethically defensible compared to the present-day practice of implementing interventions based on weak evidence [9, 25, 44, 45, 52, 79]. We argue that the advantages of the procedure outweigh the disadvantages. The health care researchers must deliver solid proof of more benefit than harms before interventions are introduced into clinical practice.

## Conclusions

If the proposed eight-step procedure is followed, this may increase the validity of assessments of intervention effects in systematic reviews.

### Ethical approval and consent

None needed.

### Standards of reporting

There are no relevant standards of reporting for this type of paper.

### Data availability

No additional data available.

## Electronic supplementary material

Additional file 1: **Example.** (DOCX 272 KB)
