# Supplementary material for: Thresholds for statistical and clinical significance in systematic reviews with meta-analytic methods
Source: BMC Med Res Methodol. 2014 Nov 21;14:120. doi: 10.1186/1471-2288-14-120 (PMC4251848; doi:10.1186/1471-2288-14-120)
Supplement: Supplementary file 1 — Additional file 1: Example. (DOCX 272 KB) [file 12874_2014_1132_MOESM1_ESM.docx]

# Example

A systematic Cochrane review by Bjelakovic et al. assessed the effects of vitamin D supplementation on mortality [[19](#_ENREF_19)]. The overall conclusion was that Vitamin D_3_ seemed to decrease mortality in elderly people. We will now apply our eight-step procedure on the results of the review regarding the effects of vitamin D_3_ on the primary outcome all-cause mortality [[19](#_ENREF_19)]. We have used the original data from the review to demonstrate how the eight-point procedure may be used in practice.

*1. Calculate and report the confidence intervals and P-values from all fixed-effect and random-effects meta-analyses. The most conservative result should be the main result.*

Our assessment: Bjelakovic et al. reported confidence intervals and *P*-values (RR 0.94 (95% CI 0.91 to 0.98); *P* = 0.002; I2 = 0; 75,927 participants; 38 trials) [[19](#_ENREF_19)]. As there were no statistical heterogeneity the results of the random-effects meta-analysis and fixed-effect meta-analysis were similar.

*(2) Explore the reasons behind substantial statistical heterogeneity by performing sensitivity analyses.*

Our assessment: There was no statistical heterogeneity.

*3. Adjust the thresholds for significance (P-values and the confidence intervals from the meta-analyses and the risks of type I error in the trial sequential analysis) according the number of primary outcomes.*

Our assessment: Two co-primary outcomes were used. The outcomes were assessed at one time point (at the end of the trial follow-up period). Our pragmatic approach (dividing 0.05 with the value halfway between 1 and the number of primary outcome comparisons) results in a threshold for significance = 0.033.

*4. Calculate and report a realistic diversity-adjusted required information size and analyse with trial sequential analysis. Report if the trial sequential monitoring boundaries for benefit, harm, or futility are crossed [*[*24*](#_ENREF_24)*,* [*25*](#_ENREF_25)*].*

Our assessment: The authors calculated a diversity-adjusted required information size based on mortality in the control group of 10%; relative risk reduction of 5% in the experimental group; type I error of 5%; and type II error of 20% (80% power). The required information size was 115,505 participants. Using the pragmatic approach to adjust the risk of type I error according to the two co-primary outcomes (see **step 3**) the risk of type I error should be lowered to 0.033 due to the use of two co-primary outcomes (see **step 3**). We have therefore calculated a slightly higher required information size of 120,200 participants (see graph below). As in the review, the boundary for benefit is still crossed indicating a statistically significant beneficial effect of vitamin D_3_:

*5. Calculate and report Bayes factor for the primary outcome (or outcomes) based on the anticipated intervention effect, which is also used to estimate the diversity-adjusted required information size. A Bayes factor less than 0.1 (a ten-fold higher likelihood of compatibility with the alternative hypothesis than the likelihood of compatibility with the null hypothesis) may be chosen as threshold for significance.*

Our assessment:

First the standard error for the ln RR is calculated based on the confidence intervals (95% CI 0.91 to 0.98):

Lower limit = ln(lower confidence limit given for RR) = ln(0.91) = -0.094

Upper limit = ln(upper confidence limit given for RR) = ln(0.98) = -0.020

Standard error = (upper limit – lower limit) / 3.92 = (-0.020) – (-0.094) / 3.92 = 0.019

And the intervention effect estimate = ln(RR) = ln (0.94) = -0.061

The anticipated intervention effect used to calculate the required information size was a RR of 0.95 ≈ ln(.95) = -0.051

Bayes factor can then be calculated using the formula (http://www.ctu.dk/tools-and-links/bayes-factor-calculation.aspx):

Bayes factor = 0.007 which is below the recommended threshold of 0.1

*(6) Use subgroup analysis and sensitivity analyses to assess the potential impact of systematic errors (bias).*

Our assessment:

**Overall risk of bias:** Including only trials with low risk of bias in the meta-analysis still shows a statistically significant meta-analysis result (*P*-value = 0.009 which is below 0.033). However, the boundary for benefit is not crossed using trial sequential analysis only including trials with low risk of bias:

Calculating Bayes factor based only on the results from trials with low risk of bias:

First the standard error for the ln RR is calculated based on the confidence intervals (95% CI 0.89 to 0.98):

Lower limit = ln(lower confidence limit given for RR) = ln(0.89) = -0.117

Upper limit = ln(upper confidence limit given for RR) = ln(0.98) = -0.020

Standard error = (upper limit – lower limit) / 3.92 = (-0.020) – (-0117) / 3.92 = 0.025

And the intervention effect estimate = ln(RR) = ln (0.93) = -0.073

The anticipated intervention effect used to calculate the required information size was a RR of 0.95 ≈ ln(0.95) = -0.051

Bayes factor can then be calculated using the formula:

Bayes factor = 0.020 which is below the recommended threshold of 0.1.

**The potential impact of missing data**

The authors only present a ’best-worst case’ scenario and a ‘worst-best case’ scenario on the overall results including trials both with low and high risk of bias. The authors conclude that these meta-analyses results show that there is a potential risk of attrition bias in this systematic review.

’Best-worst case’ scenario including only trials with low risk of bias assessing the effects of vitamin D_3_: when it is assumed that participants lost to follow-up in the experimental intervention group survived and all those with missing outcomes in the control intervention group died, vitamin D significantly decreased mortality (RR 0.50 (95% CI 0.39 to 0.64); *P* < 0.00001).

‘Worst-best case’ scenario including only trials with low risk of bias assessing the effects of vitamin D_3_: when it is assumed that all participants lost to follow-up in the experimental intervention group died and all those lost to follow-up in the control intervention group survived, vitamin D significantly increased mortality (RR 2.05 (95% CI 1.61 to 2.61); *P* < 0.00001).

We can conclude (as the authors did in the overall analyses) that these meta-analyses results show that there is a risk of attrition bias in this systematic review.

To assess the uncertainty of the estimation of the anticipated intervention effects we calculated Bayes factor based on a relative risk halfway between the anticipated intervention effect used in the calculation of the required information size and 1.0:

RR of 0.975 ≈ ln(0.975) = -0.025 ≈ Bayes factor = 0.089 which is below the recommended threshold of 0.1.

*7. Assess the risk of publication bias (funnel plot).*

Our assessment: The authors published five funnel plots as supplementary material (http:// ctu.dk/publications/supplementary-material.aspx). Inspection of the funnel plots does not suggest potential bias. Furthermore, the adjusted rank correlation test (P = 0.44) and the regression asymmetry test (P = 0.08) found no statistically significant evidence of bias.

*8. Assess and report clinical significance of the results if the prior seven steps of the eight-step procedure have shown indications of a statistically significant result.*

It can be discussed whether clinical significance should be assessed when the boundary for benefit in the trial sequential analysis is not crossed when only trials with low risk of bias are included in the analysis and when there is a high risk of attrition bias.

If clinical significance is assessed for demonstration purposes, presenting number-needed-to-treat may help to demonstrate the potential impact of the effects of vitamin D_3_. Number-needed-to-treat can be calculated based on the observed RR (RR 0.94) and an assumed risk in the control group (in the calculation of the required information size it was assumed that 10% (0.1) in the control group would die):

Number-needed-to-treat = 1 / (assumed control group risk * (1 – RR)) = 1 / ((0.1 * (1 - 0.94)) = 167 participants.

Conclusion: The boundary for benefit is not crossed in the trial sequential analysis if only trials with low risk of bias are included. However, the possible bias effect seems absent as the trials with high risk of bias estimate a lower beneficial effect than the trials with low risk of bias. This might indicate that the lack of statistical significance might be due to lack of power. There seems to be a large risk of attrition bias. Otherwise, the review results show a statistically significant effect. The potential clinical population impact of vitamin D_3_ could be very large, but needs confirmation in new randomised placebo-controlled trials with maximal efforts to reduce risk of attrition bias as well as other bias risks.
